# Supplementary material for: Cyclic Electron Flow-Coupled Proton Pumping in Synechocystis sp. PCC6803 Is Dependent upon NADPH Oxidation by the Soluble Isoform of Ferredoxin:NADP-Oxidoreductase
Source: Microorganisms. 2022 Apr 21;10(5):855. doi: 10.3390/microorganisms10050855 (PMC9144156; doi:10.3390/microorganisms10050855)
Supplement: Supplementary file 1 [file microorganisms-10-00855-s001.zip › microorganisms-1641749-supplementary.pdf]

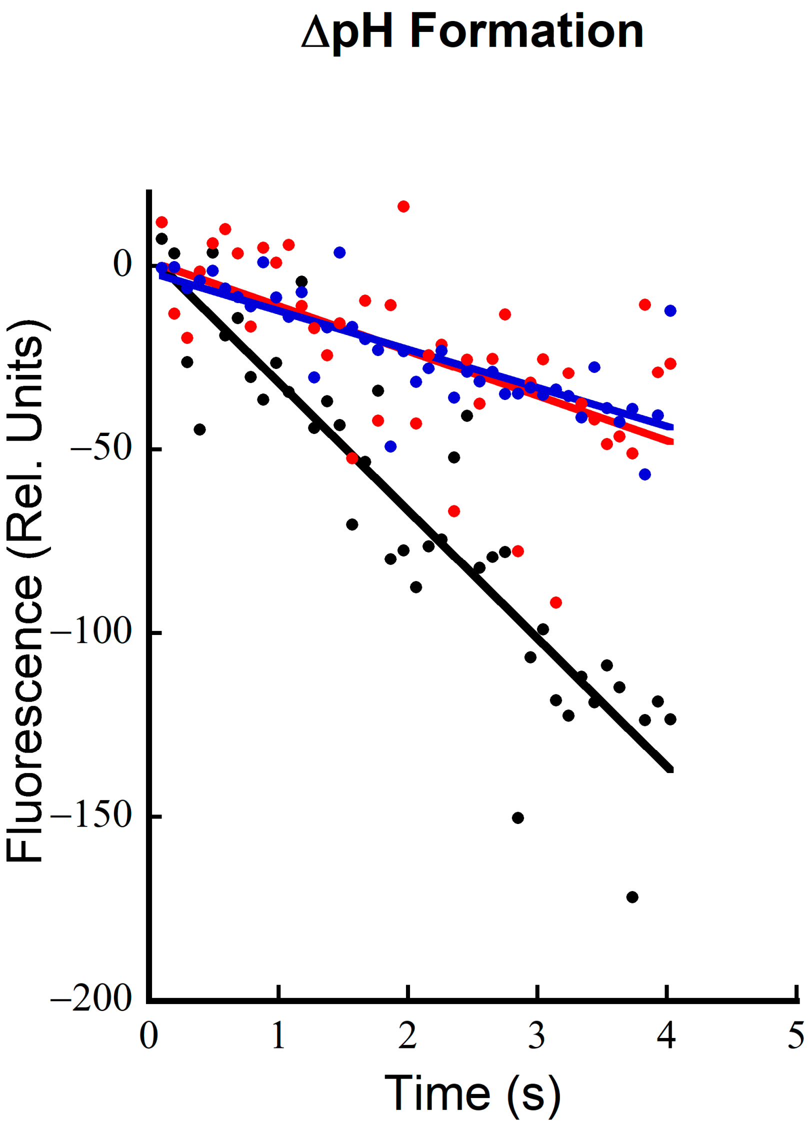

Supplementary Figure S1. Dependence of proton pumping rate on sodium gradient. WT cells in a sodium-containing buffer when treated with the sodium gradient disruptor monensin have acridine orange fluorescence quenching rates similar to cells in a buffer lacking sodium. Acridine orange fluorescence of dark-adapted WT cells in the JTS-100 in response to the presence of monensin in TCN buffer. Cells were dark adapted with acridine orange (5µM) for 20 min in either TCN buffer (black, red) or TCK buffer (blue). For samples in TCN buffer, a sample was prepared with KCl (150mM) plus Val (10µM), DCCD (500µM), and KCN (200µM) and stirred in the dark for 5 min (black trace) or with the addition of 50µM monensin (red). A sample in TCK buffer with the addition of Val (10µM), DCCD (500µM), and KCN (200µM) was otherwise treated the same as the black trace (blue trace).
